# Investigating an outbreak of measles in Margibi County, Liberia, October 2015

**DOI:** 10.11604/pamj.supp.2017.27.1.12564

**Published:** 2017-05-28

**Authors:** Joseph Asamoah Frimpong, Maame Pokuah Amo-Addae, Peter Adebayo Adewuyi, Meeyoung Mattie Park, Casey Daniel Hall, Thomas Knue Nagbe

**Affiliations:** 1 Liberia Field Epidemiology Training Program, Monrovia, Liberia; 2 Rollins School of Public Health, Emory University, Atlanta, USA; 3 Ministry of Health, Monrovia, Liberia

**Keywords:** Public health, epidemiology, outbreak investigation, infectious disease, re-emerging

## Abstract

The emergence and re-emergence of infectious diseases highlights the need to have well-trained field epidemiologists who will be at the forefront in the fight against these diseases, especially during an outbreak. Training for outbreak investigation is most effective when participants can develop their competencies in a practical exercise. To that end, this case study was based on a measles outbreak investigation conducted in Liberia during October 2015 by Liberia Frontline Field Epidemiology Training Program (FETP) residents, simulating steps to perform outbreak investigation in a real-life situation as a field epidemiologist. This case study is ideally suited to reinforce principles and skills already covered in a classroom lecture or in background reading by providing a practical training beyond the scope of theoretical learning. It is primarily intended for training novice public health practitioners who should be able to complete the exercises in approximately 3 hours.

## How to use this case study

**General instructions:** ideally, 1 to 2 instructors facilitate the case study for 8 to 20 students in a classroom or conference room. The instructor should direct participants to read a paragraph out loud, going around the room to give each participant a chance to read. When the participant reads a question, the instructor directs all participants to perform calculations, construct graphs, or engage in discussions. The instructor may split the class to play different roles or take different sides in answering a question. As a result, participants learn from each other, not just from the instructors. Specific instructor’s notes are included with each question in the instructor’s version of this case study.

**Audience:** residents in Frontline Field Epidemiology Training Programs (FETP-Frontline), Field Epidemiology and Laboratory Training Programs (FELTPs), and others who are interested in this topic.

**Prerequisites:** before using this case study, participants should have received lectures or other instruction in outbreak investigation.

**Materials needed:** laptop with Microsoft Excel or graph paper, flipchart or white board with markers

Level of training and associated public health activity: Novice - Outbreak investigation

**Time required:** approximately 3 hours

**Language:** English

## Case study material

- Download the case study student guide (PDF - 2.36 MB)
- Request the case study facilitator guide
